# Dynamics of Plasmatic Levels of Pro- and Anti-Inflammatory Cytokines in HIV-Infected Individuals with *M. tuberculosis* Co-Infection

**DOI:** 10.3390/microorganisms9112291

**Published:** 2021-11-04

**Authors:** Marina Nosik, Konstantin Ryzhov, Irina Rymanova, Alexandr Sobkin, Alexey Kravtchenko, Ulyana Kuimova, Vadim Pokrovsky, Vitaly Zverev, Oxana Svitich

**Affiliations:** 1 I.I. Mechnikov Institute of Vaccine and Sera, 105064 Moscow, Russia; rkazaw@yahoo.com (K.R.); vitalyzverev@outlook.com (V.Z.); svitichoa@yandex.ru (O.S.); 2 G.A. Zaharyan Moscow Tuberculosis Clinic, Department for Treatment of TB Patients with HIV Infection, 125466 Moscow, Russia; rimanov.81@mail.ru (I.R.); alexandr@sobkin.net (A.S.); 3 Central Research Institute of Epidemiology, 111123 Moscow, Russia; alexey-kravtchenko@yandex.ru (A.K.); ulyanakuimova@gmail.com (U.K.); pokrovsky.vad@yandex.ru (V.P.)

**Keywords:** HIV, tuberculosis, HIV/TB co-infection, cytokine profile, ART, immune reconstitution inflammatory syndrome

## Abstract

Tuberculosis (TB) and HIV have profound effects on the immune system, which can lead to the activation of viral replication and negatively regulate the activation of T cells. Dysregulation in the production of cytokines necessary to fight HIV and *M. tuberculosis* may ultimately affect the results of the treatment and be important in the pathogenesis of HIV infection and TB. This work presents the results of a study of the expression of pro- and anti-inflammatory cytokines (IFN-γ, TNF-α, IL-2, IL-4, IL-6, IL-10, IL-1RA) in drug-naïve patients with dual infection of HIV/TB at the late stages of HIV-infection, with newly diagnosed HIV and TB, and previously untreated HIV in the process of receiving antiretroviral (ART) and TB treatment vs. a cohort of patients with HIV monoinfection and TB monoinfection. The study revealed that during a double HIV/TB infection, both Th1 and Th2 immune responses are suppressed, and a prolonged dysregulation of the immune response and an increased severity of the disease in pulmonary/extrapulmonary tuberculosis is observed in HIV/TB co-infection. Moreover, it was revealed that a double HIV/TB infection is characterized by delayed and incomplete recovery of immune activity. High levels of IL-6 were detected in patients with HIV/TB co-infection before initiation of dual therapy (2.1-fold increase vs. HIV), which persisted even after 6 months of treatment (8.96-fold increase vs. HIV), unlike other cytokines. The persistent enhanced expression of IL-6 in patients with dual HIV/TB co-infection allows the consideration of it as a potential marker of early detection of M. tuberculosis infection in HIV-infected individuals. The results of multivariate regression analysis showed a statistical trend towards an increase in the incidence of IRIS in patients with high IL-1Ra levels (in the range of 1550–2500 pg/mL): OR = 4.3 (95%CI 3.7–14.12, *p* = 0.53), which also allows IL-1Ra to be considered as a potential predictive biomarker of the development of TB-IRIS and treatment outcomes.

## 1. Introduction

Currently, it is well known that both HIV and tuberculosis (TB) have a profound effect on the immune system and are characterized by a dysregulation of the normal balance of cytokines and the functioning of the cytokine network. In the pathogenesis of these infections, the imbalance of cytokines produced by Th1 and Th2 lymphocytes and macrophages occupies a central place, affecting the strength of the immune system’s response to specific pathogens [1,2,3]. The imbalance of cytokine secretion in HIV infection affects the function of the immune system and the course of the disease, increasing or suppressing viral replication [4,5]. Numerous data indicate that, despite effective antiretroviral therapy (ART), there is evidence of persistent viral production and persistent immune activation in HIV-infected individuals [6,7,8]. It has been shown that persistent immune activation during viral load suppression correlates with a high risk of developing concomitant diseases not related to AIDS, including metabolic syndrome [9,10,11,12], cardiovascular diseases [9,13,14,15], neurocognitive disorders [16,17,18,19], and malignant neoplasms [20,21,22,23] and is also directly associated with increased mortality [24,25].

Immunosuppression caused by HIV-1 can also be aggravated by persistent *Mtb (M. tuberculosis)* infection. The first line of defense against *Mtb* is alveolar macrophages, which are capable of suppressing bacterial growth through phagocytosis, as well as participating in a wide range of reactions of cellular anti-tuberculosis immunity [26]. However, if *Mtb* manages to disrupt the autophagy process via various mechanisms, then macrophages become a reservoir for slow *Mtb* replication and then a reservoir for the persistent infection inside the phagosome in the latent form of TB [27,28]. As a result, immune activation is observed both in the active form of TB and in the latent form [29].

It is generally known that cytokines are significant parts of the immune response. Different cytokines have biologically overlapping functions and can regulate the production of other cytokines [30,31,32]. Therefore, the analysis of cytokines expressed during a double HIV/TB infection while patients receive ART may be important for predicting the course of the disease and the success of the treatment. The determination of plasma cytokine concentration levels can provide clinicians with prognostic markers for monitoring the damage/recovery of the immune system. At present, the production of various cytokines in HIV/TB co-infection is still insufficiently studied. There are few works devoted to the problem of studying the cytokine profile in HIV patients in the process of receiving ART. There is almost no work on the study of the cytokine status in patients with a combined HIV/TB infection before and in the process of receiving dual therapy—antiretroviral and anti-tuberculosis. However, for understanding the pathogenesis of a combined infection and effective treatment, it is vital to accurately know how deeply disturbed the production of various cytokines in patients with HIV/TB. The goal of this work was to study the expression of a number of pro-inflammatory and anti-inflammatory cytokines in patients with double HIV/TB infection, newly diagnosed with HIV and TB, and previously untreated HIV before and during the process of receiving antiretroviral/anti-tuberculosis therapy and to identify possible potential predictive markers that allow clinicians to foresee the development of the disease and evaluate the effectiveness of therapy.

## 2. Materials and Methods

### 2.1. Study Group

The subjects were recruited from three different population pools at two large medical centers: the G.A. Zaharyan Moscow Tuberculosis Clinic and Central Research Institute of Epidemiology: patients with dual HIV/TB infection, HIV-positive patients, patients with TB, and healthy controls. Patients with HIV/TB were naïve for ART and anti-tuberculosis therapy, HIV-positive patients were naïve for ART, and TB patients were naïve for anti-tuberculosis therapy. TB diagnoses were based on clinical symptoms, sputum microscopy and radiological analyses. The patients were diagnosed as HIV-seropositive by ELISA and confirmed by Western blot. Healthy controls from the general population that were seronegative for HIV and without any history of TB or exposure to the disease within the past 6 months were recruited for the study.

All patients with HIV-monoinfection were newly diagnosed with HIV. In a cohort of patients with HIV/TB, 40.3% of the patients were aware of their HIV-positive status before admission to the hospital, were drug-naïve and were newly diagnosed with TB. Moreover, 59.7% of the patients with HIV/TB were newly diagnosed with HIV and TB. All patients with TB alone were newly diagnosed with TB. The ART for patients with HIV-monoinfection was started immediately after admission to the hospital, as well as anti-tuberculosis treatment (ATT) for patients with TB-monoinfection. As for HIV/TB patients, ATT was started after admission to the hospital and ART was started within 2–3 weeks after the initiation of TB therapy. The ART regimen was: 2 NRTIs + 1 NNRTI (74.5%) and 2 NRTIs + 1 IP (25.5%). For the treatment of TB, first-line drugs were used (isoniazid, rifampicin, streptomycin, and ethambutol). No differences in the expression of cytokines were observed for diverse therapy regimens.

Patients who developed inflammatory immune recovery syndrome (IRIS) after starting the therapy were excluded from the study, as these patients represented a special group characterized by an atypical manifestation of clinical and immunological indicators. On average, the percentage of cases of IRIS in HIV/TB co-infection is relatively low, so it seemed more important to study the expression of cytokines in a group of HIV/TB patients who did not develop IRIS and who represent the majority of patients in percentage terms. The only exception was made when quantifying the levels of IL-1RA given the extreme importance that this cytokine plays in the development and course of TB, in particular in restraining and preventing the spread of infection by the formation of granulomas. In patients who developed IRIS, ART was temporarily interrupted, and the period of ATT without antiretroviral drugs was prolonged up to 12–15 weeks. At the end of this period, antiretroviral therapy was again resumed. The patients were monitored for up to 6 months. Blood samples were collected prior to the commencement of ART/anti-tuberculosis therapy and 30, 45–75, and 180 days after the initiation of treatment. Plasma was separated from the whole blood samples and stored at –80 °C until further analysis.

### 2.2. Ethical Aspects

All patients were over 18 years old and gave written informed consent for participation in the study. According to the General Data Protection Regulation (GDPR) requirements, all participants were de-identified and anonymized by assigning them unique codes expressed as an identifier. All clinical samples, data and study results were stored in an anonymized form. The study was conducted according to the guidelines of the Declaration of Helsinki and approved by the Biomedical Ethics Committee of the I.I. Mechnikov Institute of Vaccines and Sera, Moscow, Russia (protocol #1/01/17/2018).

### 2.3. Sputum Microscopy and Culture

Sputum samples were stained for acid-fast bacilli and were graded by light microscopy. Cultures were examined weekly until positive for visible colonies or for a maximum of 8 weeks.

### 2.4. CD4+ T-CELL Count

The CD4+ T-CELL count was carried out according to a standard procedure. The CD4+ T cell counting was performed by two-color flow cytometry using phycoerythrin-labeled anti-CD4 antibodies (FACSort, Becton Dickinson, Franklin Lakes, NJ, USA) according to the manufacturer’s instructions. The whole blood sample with anticoagulant was incubated with the fluorescent antibodies, and then the CD4+ cell number was determined by flow cytometry using Fluorescent Activated Cell Sorter BD FACSCount TM system (Becton Dickinson).

### 2.5. Cytokine Quantitation

All cytokines were measured before the initiation of therapy in all studied groups. In patients with HIV alone—before initiation of ART; in patients with TB alone—before initiation of ATT. The cytokines in the cohort of patients with HIV/TB were measured before the start of dual therapy—antiretroviral, as well as anti-tubercular treatment. Afterwards the cytokines were measured at 30, 60, 75–90 and 150–180 days after initiation of treatment. Plasma levels of the cytokines IL-1Ra, IL-2, IL-4, IL-6, IL-10, IFN-γ, and TNF-α were measured using ELISA EIA-BEST Kit (Vector-Best, Moscow, Russia, RF). Plasma was isolated according to the standard procedure. The whole blood was collected in a vacutainer with EDTA and centrifuged at 1000 rmp for 15–20 min. Then the plasma was aliquoted and stored at −80 °C until further analysis. The cytokine concentrations were determined using a standard curve obtained with the standards provided by the manufacturer with each kit (sensitivity 0–5 pg/mL), and the results were expressed as pg/mL.

### 2.6. TB-IRIS Definition

Paradoxical TB-IRIS was defined as the presence of at least one major or two minor TB-IRIS clinical criteria according to the International Network for the Study of HIV-Associated IRIS case definition (INSHI) [33]. Major TB-IRIS criteria were new or worsening (1) lymph nodes, cold abscesses, or other focal tissue involvement; (2) radiologic features of TB; (3) central nervous system TB; or (4) serositis (pleural effusion, (pleural effusion, ascites, or pericardial effusion). Minor TB-IRIS criteria were new or worsening: (1) constitutional symptoms (fever, night sweats, or weight loss), (2) respiratory symptoms (cough, dyspnea), and (3) abdominal pain accompanied by peritonitis, hepatomegaly, splenomegaly, or abdominal adenopathy [33]. Lymph node TB-IRIS was defined by enlarged peripheral lymph nodes on clinical examination, enlarged thoracic nodes on a chest radiograph, or enlarged abdominal nodes on an ultrasound or CT scan. Abdominal TB-IRIS was defined by abdominal symptoms attributed to TB-IRIS.

### 2.7. Statistical Analysis

The nonparametric Mann–Whitney test was performed to check for the significant differences. A *p*-value < 0.05 was considered significant. Multivariate regression analysis was employed in order to evaluate the incidence of TB-IRIS in patients with HIV/TB co-infection. Data analysis was performed using IBM SPSS Statistics 17.0.

## 3. Results

A total of 356 people participated in the study: 116 patients with HIV/TB co-infection (11 patients were subsequently excluded from the study due to non-compliance with the treatment regimen and 16 patients who developed IRIS), 80 patients with HIV monoinfection, 78 patients with TB monoinfection, and 82 healthy controls. Baseline demographic and clinical characteristics of the patients are presented in Table 1. The groups did not differ significantly in demographic indicators. Men predominated in all groups, and the median age was 37.8 years for men and 39.7 years for women. The median CD4+ cell count in the group of patients with double infection was 114 cells/mm^3^, and the viral load was 6.8 log_10_ copies/mL. Patients with severe immunosuppression prevailed in this group (71.9%). In patients with HIV alone, the viral load was approximately the same as in the HIV/TB co-infection group (6.2 log_10_ copies/mL), but the percentage of patients with severe immunosuppression was lower (40.3%). Disseminated tuberculosis prevailed among patients with HIV/TB (46.1%), and infiltrative tuberculosis prevailed among patients with TB only (67.9%). There were no fatal outcomes among the patients recruited in the study, and in the process of treatment in the study group, there were no patients who developed MDR- or XDR-TB.

### 3.1. Expression of Cytokines before ART

**Figure 1:**
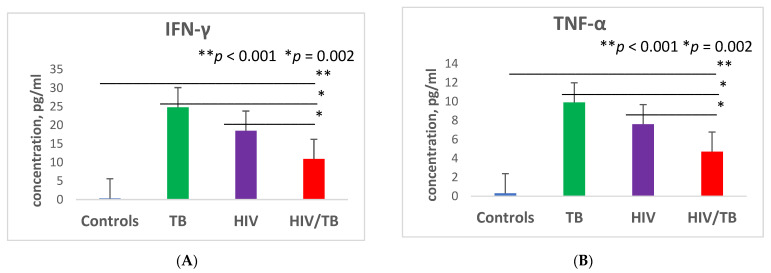

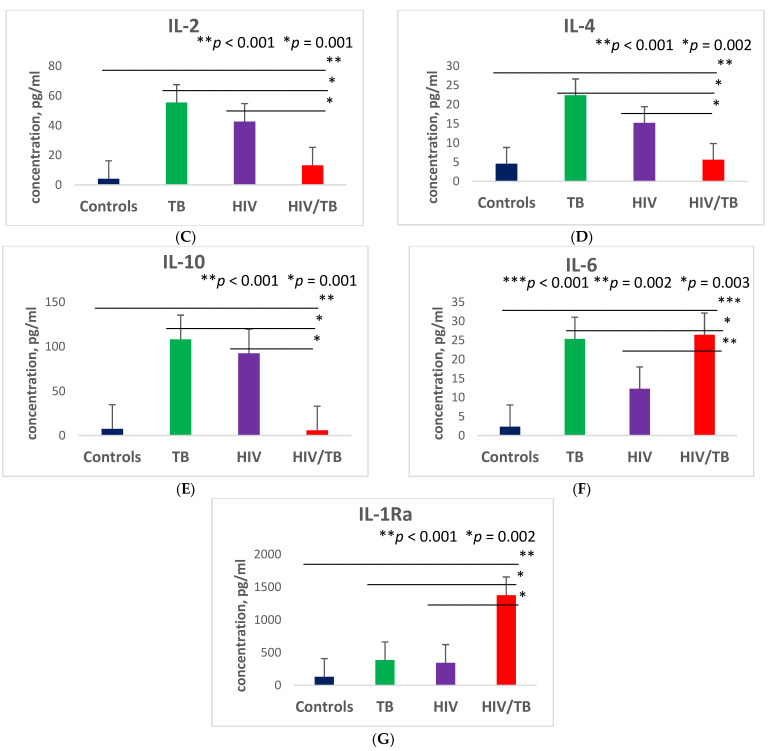
Plasma levels of different cytokines in patients with dual HIV/TB infection, HIV monoinfection, TB monoinfection, and healthy controls before therapy: interferon-γ (IFN-γ) (**A**), tumor necrosis factor-α (TNF-α) (**B**), interleukin-2 (IL-2) (**C**), interleukin-4 (IL-4) (**D**), interleukin-6 (IL-6) (**E**), interleukin-10 (IL-10) (**F**), and interleukin-1 receptor antagonist (IL-1RA) (**G**). Bars represent standard deviation.

The group of patients with combined HIV/TB infection before the start of dual therapy (anti-tubercular and antiretroviral) had reduced expression of the Th1 cytokines interferon-γ (IFN-γ), tumor necrosis factor-α (TNF-α), and interleukin-2 (IL-2) compared with the group of patients with HIV monoinfection and TB monoinfection (Figure 1). There was a 1.7-fold decrease of INF-γ production in patients with HIV/TB co-infection compared to the group of patients with HIV monoinfection and a 2.3-fold decrease in comparison with the group of patients with TB monoinfection (*p* = 0.002 and *p* = 0.002, respectively). TNF-α secretion was reduced in patients with HIV/TB against patients with HIV alone by 1.6 times (*p* = 0.002) and against patients with TB only by 2.1 times (*p* = 0.002).

The concentration of plasma IL-2 in patients with double infection was reduced by 3.3 times compared to the group of patients with HIV monoinfection (*p* = 0.001) and by 4.2 times compared to the group with TB monoinfection (*p* = 0.001). Altogether, the production of IFN-γ, TNF-α, and IL-2 in patients with HIV/TB, HIV, and TB was higher compared with the group of healthy controls (*p* < 0.001; *p* < 0.001; *p* < 0.001, respectively).

Furthermore, in patients with HIV/TB, a reduced expression of Th2 class cytokines was detected before the initiation of dual therapy compared to the groups of patients with HIV monoinfection and TB monoinfection (Figure 1). Thus, the concentration of interleukin-4 (IL-4) was reduced in the group of patients with HIV/TB compared with the group of patients with HIV by 2.7 times (*p* = 0.002) and by 4 times with the group of patients with TB (*p* = 0.002). The level of interleukin-10 (IL-10) production in the HIV/TB group was reduced 15.6 times compared to the HIV group (*p* = 0.001) and 21.6 times (*p* = 0.001) compared to the HIV group. The level of IL-10 in patients with HIV/TB was even lower than in the control group (*p* < 0.001).

A different pattern was observed in the expression of the cytokines interleukin-6 (IL-6) and interleukin-1 receptor antagonist (IL-1Ra) (Figure 1). Prior to the start of therapy, IL-6 secretion in patients with HIV/TB was increased by 2.1 times compared to the group of patients with HIV alone (*p* = 0.002) and was actually on the same level as in the group of patients with TB monoinfection (*p* = 0.003). IL-1Ra secretion was also significantly increased compared with patients with HIV monoinfection by 4 times (*p* = 0.002) and compared with patients with TB monoinfection by 3.6 times (*p* = 0.002).

There was no correlation between the level of cytokine expression, viral load, and the number of CD4 cells.

### 3.2. Expression of Cytokines in the Process of Treatment

**Figure 2:**
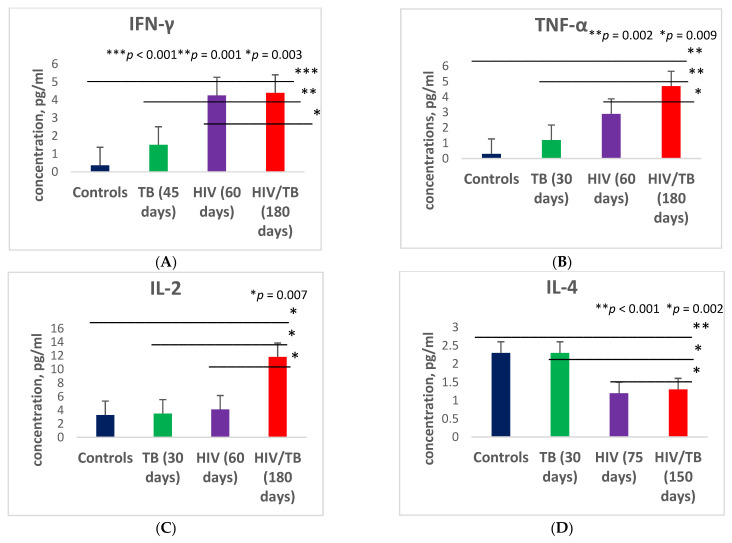

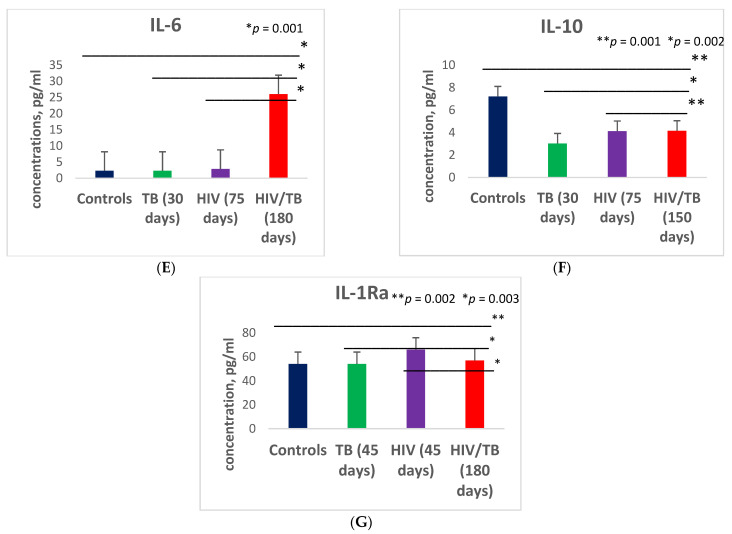
Plasma levels of different cytokines in patients with dual HIV/TB infection, HIV monoinfection, TB monoinfection, and healthy donors after dual therapy: interferon-γ (IFN-γ) (**A**), tumor necrosis factor-α (TNF-α) (**B**), interleukin-2 (IL-2) (**C**), interleukin-4 (IL-4) (**D**), interleukin-6 (IL-6) (**E**), interleukin-10 (IL-10) (**F**), and interleukin-1 receptor antagonist (IL-1RA) (**G**). Bars represent standard deviation.

**Figure 3:**
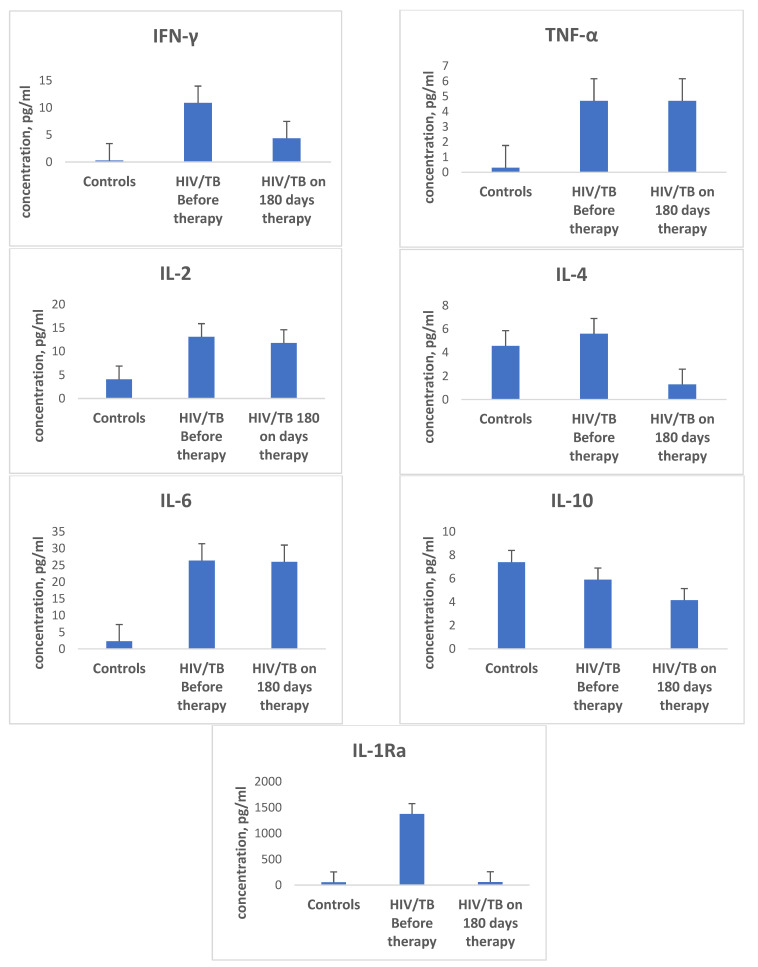
Plasma levels of different cytokines in patients with dual HIV/TB infection before and after dual therapy (therapy duration 180 days): interferon-γ (IFN-γ), tumor necrosis factor-α (TNF-α), interleukin-2 (IL-2), interleukin-4 (IL-4), interleukin-6 (IL-6), interleukin-10 (IL-10), and interleukin-1 receptor antagonist (IL-1RA). Bars represent standard deviation.

If patients with HIV/TB showed a significant decrease in the secretion of most Th1 and Th2 class cytokines before the start of therapy (antiretroviral and anti-tubercular), the observation of this group of patients in the process of receiving therapy showed that, in general, in patients with HIV/TB co-infection, the levels of most pro- and anti-inflammatory cytokines in the blood plasma were significantly higher during the 180 days of treatment than in the control group and in the group of patients with HIV only or only with TB (Figure 2 and Figure 3).

At the same time, in patients with HIV monoinfection and TB monoinfection, a level of cytokine secretion comparable to similar indicators in the group of healthy controls was already observed, on average, at 30–60 days of treatment. Thus, in the group of patients with TB alone, a level of expression of IL-2 and TNF-α comparable to similar indicators in the control group was reached on the 30th day after the start of therapy, and in the HIV group, this was achieved on the 60th day after the initiation of therapy. In the group of patients with combined HIV/TB infection, on the 180th day of therapy, the concentration of IL-2 increased by an average of 3.3 times compared to the other three groups (*p* = 0.007), and the concentration of TNF-α exceeded 15.7 times the level of the same indicator in the control group, 3.9 times in the group with TB alone, and 1.6 times in the group of patients with HIV monoinfection (*p* = 0.009; *p* = 0.02 and *p* = 0.02, respectively). The levels of expression of IL-4 and IL-10 comparable to those for similar indicators in the control group in patients with TB were achieved after 30 days of therapy, while in patients with HIV, this was achieved after 75 days of therapy, and in the HIV/TB group, this was achieved only on the 150th day of therapy. For IFN-γ and IL-1Ra, a secretion level comparable to similar indicators in the control group was reached after 180 days of therapy.

The exception was the cytokine IL-6. On the 180th day after therapy, the level of IL-6 expression in the HIV/TB group was 11.3 times higher compared to the control group and the TB group, and 8.96 times higher compared to the group of patients with HIV monoinfection (*p* = 0.001; *p* = 0.001; *p* = 0.001, respectively), actually remaining at the same level as before the initiation of therapy.

Additionally, before the start of ART, in the process of receiving therapy, neither in patients with HIV/TB nor in patients with HIV alone was there a significant correlation between the level of cytokine expression, viral load, and the number of CD4 cells.

### 3.3. Expression of Cytokines Depending on the CD4+ Cell Count

**Figure 4:**
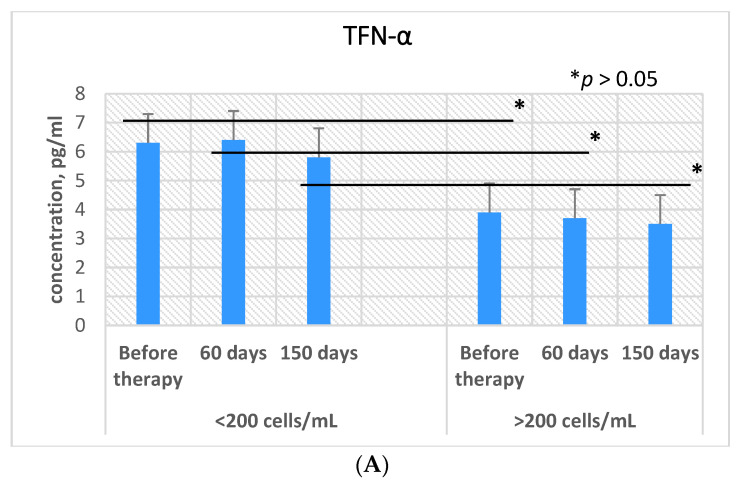

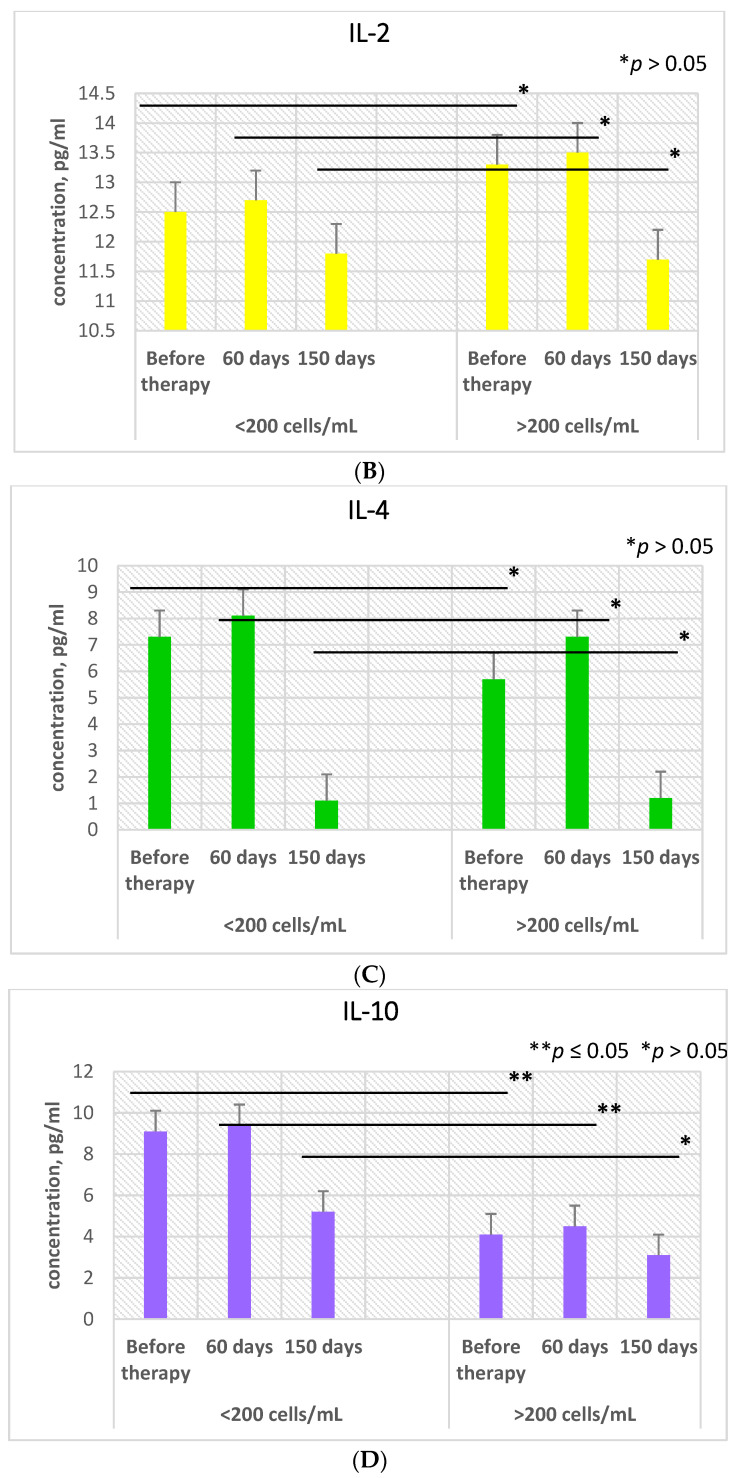

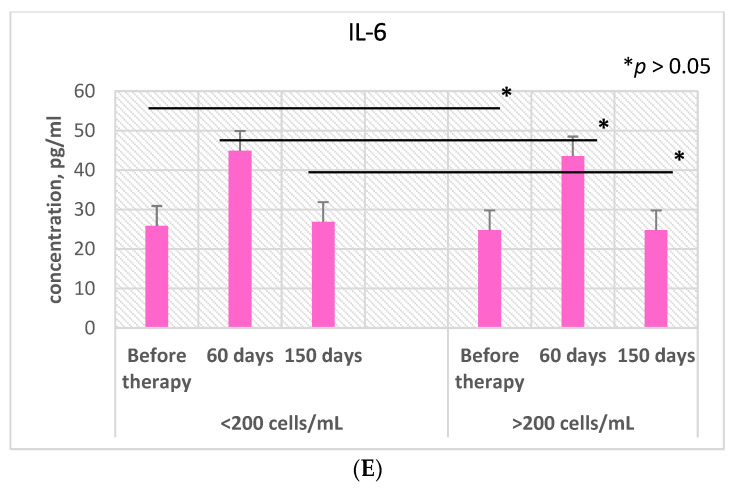
Plasma levels of different cytokines in patients with dual HIV/TB infection depending on the CD4 cell count: tumor necrosis factor-α (TNF-α) (**A**), interleukin-2 (IL-2) (**B**), interleukin-4 (IL-4) (**C**), interleukin-10 (IL-10) (**D**), and interleukin-6 (IL-6) (**E**). Bars represent standard deviation.

In order to identify a possible correlation of the level of cytokine expression with the number of CD4+ cells, the secretion of some pro- and anti-inflammatory cytokines was studied in a group of patients with CD4+ < 200 and CD4+ > 200 cells/mm^3^ (Figure 4).

In a comparative study of the level of production of IL-2, IL-4, and IL-6 between the two groups, practically no difference was found, both before therapy and on the 180th day of receiving therapy. At the same time, an increase in the expression of IL-6 in both groups was detected by 1.7 times on the 60th day after the start of therapy (*p* = 0.003). However, on the 180th day of therapy, the secretion of IL-6 decreased and remained at the same level as before therapy.

Lower levels of TNF-α and Il-10 production were observed in the group of patients with CD4+ >200 cells/mm^3^ compared to the group of CD4+ <200 cells/mm^3^: for TNF-α, the expression level was 1.6 times below the other group (*p* = 0.03), and for IL-10, the value was 2.2 times below the other group (*p* = 0.009).

### 3.4. Expression of the Interleukin-1 Receptor Antagonist (IL-1Ra)

IL-1Ra is involved in the natural compensatory mechanism in IL-1-induced pathological processes and is a marker of the acute phase of inflammation. In patients with active pulmonary tuberculosis, local inflammation in the lower respiratory tract can cause an increased release of IL-1Ra, so the level of this pro-inflammatory cytokine may be more representative of lung diseases [34].

Presumably, the level of this cytokine may correlate with the clinical course of the disease, such as the degree of lung damage, fever, and weight loss. Taking into account the important role of IL-1Ra in *M. tuberculosis* infection, we decided to study the expression of this cytokine in more detail in a double HIV/TB infection. Therefore, patients who developed IRIS after 1.5–2 months of ART initiation (*n* = 16/15.2%) were also included in the study. The symptoms observed in HIV/TB patients who developed IRIS are presented in Table 2.

**Figure 5:**
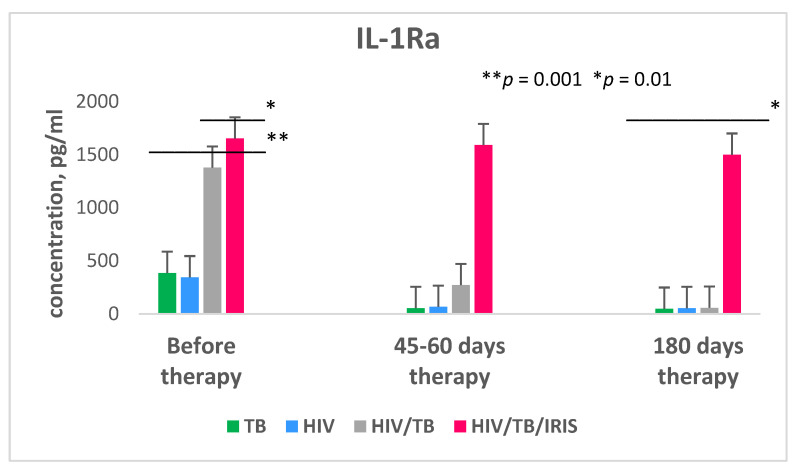
Plasma levels of interleukin-1 receptor antagonist (IL-1Ra) in patients with dual HIV/TB infection with and without immune reconstitution inflammatory syndrome (IRIS), HIV monoinfection, and TB monoinfection before and in the process of receiving therapy. For patients with HIV monoinfection before initiation of ART. For patients with TB monoinfection before initiation of ATT. For patients with HIV/TB before initiation of ART and ATT. Bars represent standard deviation.

Generalized lymphadenopathy prevailed among the systemic symptoms that developed with IRIS (43.8%). The average duration of IRIS development after the start of ART was 75.02 (±19.14) days in 62.5% (10) of patients and 119.01 (±14.17) days in 37.5% (6) of patients. When studying the expression level of IL-1Ra, it was found that in a group of patients with HIV/TB co-infection without IRIS, this indicator was on average almost four times (*p* = 0.001) higher than in patients with HIV and TB only, but it significantly decreased after 1.5–2 months of double therapy (Figure 5).

After 6 months of therapy, the production of IL-1Ra in patients with HIV/TB without IRIS reached a level comparable to that in the group of patients with only HIV and with only TB (*p* = 0.002). In patients with HIV/TB co-infection who subsequently developed IRIS, the level of IL-1Ra expression before the initiation of ART was 4.8 times higher compared to the group of patients with TB monoinfection (*p* = 0.001) and almost 1.2 times higher than the same indicator compared to the group of patients with HIV/TB co-infection without IRIS (*p* = 0.01). At the same time, this indicator remained high throughout the entire period of therapy in patients with HIV/TB who developed IRIS. After the 180 days of therapy, the level of IL-1Ra expression in this group of patients was, on average, 28.5 times higher compared with other groups of patients (*p* = 0.001). The results of multivariate regression analysis showed a statistical trend towards an increase in the incidence of IRIS in patients with high IL-1Ra levels (in the range of 1550–2500 pg/mL): OR = 4.3 (95%CI 3.7–14.12, *p* = 0.53). It should be noted that patients with double HIV/TB infection, both with and without IRIS, initially did not differ significantly in the number of CD4+ cells and viral load (Table 3). After 6 months of therapy, in general, there were also no significant differences in these indicators (Table 3). The group of patients with IRIS had a slightly lower increase in CD4+ cells, but this was not statistically significant. Furthermore, no correlation was detected between the risk of developing TB-IRIS with forms of TB or with HIV transmission route (it is assumed that drug addiction may also be one of the factors of the development of IRIS).

## 4. Discussion

Interferon-γ (IFN-γ) and TNF-α play a key role in the anti-*Mtb* cytokine cascade, as they are associated with the formation and maintenance of granulomas [35,36]. Cells recruited into an infected lung control the infection by producing IFN-γ in response to *Mtb* [37]. In patients with mutations in the genes encoding the synthesis of IFN-γ and its receptor, violations in the formation of granulomas, a decrease in the production of IFN-γ, and a pronounced depression of antimycobacterial immunity are detected [38,39]. Interferon-γ is also involved in the activation of macrophages and the induction of MHC-II synthesis, which leads to an increase in the functional activity of antigen-presenting cells, the induction of T-helpers, and an increase in the cytotoxicity of monocytes, leading to the accumulation of CD4+ T-lymphocytes and/or cytotoxic T-lymphocytes [40,41]. It has been shown that IFN-γ reduces viral replication in infected macrophages [42], and dysregulation of its expression directly correlates with the progression of immunodeficiency [43,44].

In this study, all groups of patients had increased secretion of IFN-γ in comparison with the control group. However, compared to the group of patients with only HIV and only with TB, the expression of IFN-γ in patients with double infection was significantly reduced. The decreased secretion of IFN-γ in HIV/TB patients revealed by us in comparison with the groups of patients with HIV monoinfection and TB monoinfection is consistent with the results of other researchers who also showed a reduced secretion of IFN-γ, both in HIV infection and in double HIV/TB infection [2,44,45,46]. Moreover, if in TB patients, this indicator became comparable with the values in the control group after 1.5 months of therapy and in HIV-positive patients on the second month of therapy, in patients with double infection, it occurred only after 6 months of therapy. Studies conducted by Osuji et al. [4] have shown that the reduced concentration of IFN-γ can remain in HIV patients, even after 12 months of therapy. Partly, this may be explained by the fact that chronic HIV infection is characterized by the depletion of Th cells secreting IFN-γ [2]. It is likely that HIV-1 infection leads to a modification of the antigen presentation in macrophages and dendritic cell lines, which in turn leads to the anergy of HIV-1-specific CD4+ and CD8+ T cells [40]. Although it is assumed that in HIV infection, the expression of IFN-γ and its immunological functions are shifted more towards pro-inflammatory action than immunoregulatory functions [40,47], it is believed that IFN-γ can affect the development of HIV infection by changing the expression of chemokine receptors and their ligands, leading to a change in the penetration of the virus into monocytic cells [42]. The inhibition of chemokine receptor expression induced by IFN-γ may limit the recruitment of monocytes to the sites of development of the pathological process in TB and HIV infection, thereby contributing to chronic inflammation [42].

Tumor necrosis factor-α (TNF-α), along with INF-γ and IL-2, plays an important role in the immunopathology of both HIV infection and TB. TNF-α is critically important for controlling bacterial growth during infection with *M. tuberculosis* due to phagocyte-activating functions and has an active role in the formation of granulomas [37,48,49,50,51,52]. It has been shown that anti-TNF-α therapy prevents the maturation of granulomas, promoting the free growth of *Mtb* [48,52]. In some cases, normal granuloma formation occurs during anti-TNF-α therapy, but nevertheless, it is not possible to control mycobacterial infection [53]. At the same time, TNF-α activates HIV replication in macrophages, T-lymphocytes, and monocytes by activating the NF-kB transcription factor [3,48,54]. In this study, an increased expression of TNF-α was noted in HIV monoinfection and TB monoinfection compared to the control group, which indicates the activation of the immune response. This is in accordance with the data of other researchers who received similar results [43,55,56,57]. However, in a double HIV/TB infection, we noted a reduced level of TNF-α production compared to other groups of patients. This does not contradict the results of researchers who have demonstrated that HIV-infected patients have a reduced expression of TNF-α in the granuloma microenvironment [53]. De Castro Cunha et al. also showed reduced concentrations of TNF-α in patients with HIV/TB compared to patients with only HIV and only with TB [52]. At the same time, in our study, in patients with HIV/TB after 6 months of ART, the level of TNF-α remained the same as before therapy. Probably, as in the case of IFN-γ secretion, this can be explained by the depletion of T cells. Wong et al. hypothesized that due to the negative impact of HIV-1 on CD4+ cells, their pool decreases and consequently, there are fewer CD4+ cells that can produce TNF-α and IFN-γ [48]. As a result, the remaining T-cells function in an enhanced mode, which ultimately leads to their depletion [48]. De Noranha et al. showed that in HIV/TB co-infection, HIV-1 affects the process of granuloma formation, disrupting the production of TNF-α, which leads to defective granuloma formation and necrosis and, as a result, *Mtb* dissemination [53].

Interleukin (IL-2), along with IFN-γ and TNF-α, also plays an important role in the maturation, proliferation, and functional activity of immunocompetent cells, which are restraining factors in the progression of both HIV infection and TB [58,59]. IL-2 activates T cells, NK cells, B cells, monocytes, macrophages, and neutrophils [60]. However, the role of IL-2 in HIV infection is ambiguous. With active T-cell proliferation, IL-2 can stimulate HIV replication in activated and proliferating T-lymphocytes [61,62]. At the same time, IL-2 reduces the expression of receptors on antigen-presenting cells, decreasing the rate of their infection with HIV-1 [63]. In addition, IL-2 reduces the apoptosis of T-lymphocytes and increases their survival [64]. In *M. tuberculosis* infection, IL-2 plays an important role in triggering the antimicrobial activity of macrophages and, together with IFN-γ and TNF-α, stimulates the ability of macrophages to kill *Mtb* [65]. We have shown that the level of IL-2 production in patients with double infection was significantly reduced compared to the groups of patients with only HIV and only TB, as well as with the control group. The reduced concentration of IL-2 in patients with HIV/TB co-infection was also demonstrated by a number of other researchers [46,66]. In this study, the level of IL-2 expression in the HIV/TB group of patients after the initiation of ART remained almost the same as before therapy, which as in the case of IFN-γ- and TNF-α secretion, is most likely due to the depletion of T cells. It is obvious that the reduced expression of INF-γ, IL-2, and TNF-α in patients with double HIV/TB infection indicates the suppression of the Th1 response.

Interleukin 4 (IL-4) is a pleiotropic cytokine produced by activated CD4+ T-lymphocytes, mast cells, and basophils and has multiple functions that modulate the immune response to various cell types [67]. In HIV infection, IL-4 differentially regulates the expression of CCR5 and CXCR4 co-receptors, which play a key role in HIV-1 infection, preventing the virus from entering the cell and its replication [68,69]. It has also been shown that the nucleotide polymorphism in the IL-4 gene (IL-4 2589T) prevents the progression of HIV infection by reducing the viral load [68]. At the same time, an increased expression of IL-4 leads to a greater susceptibility to *Mtb* infection [70,71]. A study conducted among medical professionals showed that those individuals whose peripheral blood mononuclear cells synthesized IL-4 in response to *M. tuberculosis* infection in vitro subsequently developed an active form of TB within 2–4 years [70,72]. Studies on Balb/c mice with IL-4 genes knocked out also proved that the absence of IL-4 leads to a decrease in *Mtb* growth [73]. Interleukin 10 (IL-10) is an anti-inflammatory cytokine secreted in response to systemic inflammation by monocytes, macrophages, T cells, and dendritic cells [74]. This cytokine inhibits the production of pro-inflammatory cytokines and the action of antigen-presenting cells, blocking the activation of T-lymphocytes by inhibiting the expression of MHC class II molecules [35]. Currently, there are conflicting data on the role of IL-10 in HIV infection. A number of researchers have shown that high levels of IL-10 inhibit the production of HIV-1 [75,76,77,78]. Conversely, other researchers have obtained contradictory data, demonstrating that an increased level of IL-10 promotes the active replication of HIV-1, inducing an enhanced expression of CCR5 and CXCR4 co-receptors [79,80]. In addition, it was found that high concentrations of IL-10 correlate with the increased expression of PD-1 (programmed death ligand-1) on monocytes [81], which leads to the depletion of T cells and the progression of HIV infection [82]. Studies conducted in vitro on human cell lines have demonstrated that blocking IL-10 can improve the immune response to HIV-1 [74]. At the same time, it was previously shown that IL-10 inhibits the apoptosis of IL-2-dependent T cells [83].

The role of IL-10 in *Mtb* infection is also not fully understood, but in vivo studies on a CBA/J mice model susceptible to *Mtb* infection have demonstrated that blocking IL-10 during chronic *Mtb* infection stabilized the pulmonary bacterial load and improved the survival of specimens [84]. The fact that high concentrations of Il-10 aggravate the course of TB infection is also confirmed by other studies in vivo on a model of transgenic mice (C57BL/6 mice model) producing an increased amount of IL-10. After infection with *M. Tuberculosis*, mice with IL-10 hypersecretion showed an accelerated progression of TB infection and signs of TB reactivation [85]. In this study, there was a significant decrease in the expression of IL-4 and IL-10 in patients with HIV/TB co-infection compared to patients with HIV monoinfection and TB monoinfection before ART. At the same time, the level of both cytokines in patients with HIV/TB was actually the same as in the control group, which indicates a deep failure of the immune system. It is assumed that, due to the persistently high viral load and constant immune activation observed in both HIV infection and TB, T-cells are depleted, and they lose their ability to function effectively [86,87]. However, in the process of receiving therapy, the level of IL-4 and IL-10 production increased slightly, and after 6 months of treatment, it reached the same level as in the group of patients with only HIV and only with TB. The reduced expression of IL-4 and IL-10 in double HIV/TB infection is in line with the earlier observations by our group [88] and by others [46]. Contradictory data were obtained by da Silva et al. and Osuji et al., who demonstrated an enhanced expression of IL-4 and IL-10 in HIV/TB co-infection [4,56]. The difference in the obtained results is probably due to the fact that the cohort of patients in our study significantly differed from the patient’s groups in other studies by clinical and immunological indicators. In this study, the majority of patients were at the late stages of HIV infection (C3–67.8%) with severe immunosuppression, and 46.1% of patients had disseminated TB, and 40.4% had infiltrative TB. Disseminated TB is a mycobacterial infection in which *Mtb* has spread from the lungs to other parts of the body through the blood or lymph system. As a result, generalized disseminated tuberculosis with foci in various organs or tuberculous sepsis develops. HIV infection also contributes to the dispersion of *Mbt* in the body. Infiltrative pulmonary tuberculosis is a secondary tuberculosis infection characterized by a widespread lesion of the lungs with an exudative type of inflammatory reaction and the formation of foci of caseous decay. In the case of further progression of infiltrative TB, two variants of development are possible: transition to caseous pneumonia or the disintegration of lung tissue with the formation of cavities. Both forms of TB in patients with late stages of HIV infection proceed with a pronounced intoxication syndrome. Thus, perhaps one of the factors explaining the reduced expression of those cytokines might be the serious condition of the patients, which influenced immunological deterioration. The revealed reduced production of IL-4 and IL-10 indicates that despite the fact that patients with HIV/TB co-infection were in the late stages of the disease, they did not have a shift towards the expression of Th2 class cytokines.

In a comparative study of the expression levels of a number of cytokines in patients with HIV/TB co-infection depending on the CD4+ cell count (CD4+ < 200 and CD4+ > 200 cells/mm^3^), there was no significant difference in the production of IL-2 and IL-4 cytokines before and after the start of therapy. The expression level of both cytokines remained almost unchanged, despite an increase in the number of CD4+ cells and a decrease in the viral load. Thus, the reduced level of expression of these cytokines is not directly associated with a decrease in the number of CD4+ cells since cell growth increased over time after the initiation of therapy. Presumably there are some other factors that affect the expression of these cytokines in patients with TB at late stages of HIV infection, which is the subject of future research. It is obvious that HIV infection and TB have an additive effect on the immune system, disrupting the secretion of cytokines. Higher levels of TNF-α and IL-10 production were observed in the group of patients with CD4+ < 200 cells/mm^3^ compared to the group with CD4+ > 200 cells/mm^3^, which indicates more intense immune activation. However, in general, in patients with combined HIV/TB infection after the start of antiretroviral/anti-tuberculosis therapy, there was a delayed and incomplete recovery of the immune activity.

IL-6 is a multifunctional cytokine with both pro-inflammatory and anti-inflammatory effects produced in response to tissue damage and infections [89,90]. The biological role of IL-6, first of all, consists of the induction of regenerative mechanisms and the activation of immune defense: the activation and differentiation of T cells, maturation of B cells, and induction of synthesis/secretion of immunoglobulins, synthesis of C-reactive protein in the liver and enhancement of hematopoiesis [91,92]. IL-6 is induced instantly at the early stages after the infection of monocytes/macrophages, with a subsequent increase in its expression [93]. Numerous data indicate that the dysregulation of IL-6 expression is the main factor in the pathogenesis of chronic inflammatory and autoimmune diseases [94,95].

The results of our study revealed that in naive patients with HIV monoinfection, as well as in patients with double HIV/TB infection, IL-6 concentration levels were significantly increased compared to the control group, which is consistent with the data obtained by other researchers [1,4,96,97,98]. At the same time, the concentration level of IL-6 in the cohort of patients with HIV/TB exceeded the same indicator in the group of patients with HIV only. The enhanced expression of IL-6 demonstrated by our group in HIV-negative patients with active TB versus the group of healthy controls is also confirmed by the results of other studies [55,99]. However, if in the cohort of HIV-positive patients, the level of IL-6 gradually decreased in the process of receiving ART and became comparable with the control group, which is consistent with the data obtained by Osuji et al. [4] and Haissman et al. [100], in this study, in patients with HIV/TB co-infection, even after 6 months of therapy, IL-6 expression remained at the same high level as before ART. The detected pronounced increase in the level of IL-6 in HIV/TB patients with severe immunosuppression in the absence of inflammatory immune recovery syndrome (IRIS) indicated less effective immune recovery, given that the median CD4+ cell count in the group with CD4+ <200 cells/mm^3^ was 57 cells/mm^3^. Evidently, such a profound dysregulation of IL-6 production in patients with HIV/TB is explained by the synergistic effect of both infections and contributes to the development of both HIV and TB pathology. Thus, it has been shown that *M. tuberculosis* induces the dysregulation of pro-inflammatory cytokines and, in particular, activates the expression of IL-6 by phagocytes [55]. The increased expression of IL-6 induced by *Mtb* leads to the inhibition of the autophagy process in infected cells [101].

In the 1990s, it was shown that the increased expression of IL-6 enhances HIV replication by a translational mechanism, which in turn leads to an increase in the viral load, the infection of still uninfected T-cells and a further decrease in the expression of Th1 class cytokines [102]. According to recent studies, the HIV-1 *vpr* regulatory gene causes the degradation of TET2 DNA demethylase, which suppresses the induction of the IL-6 gene in HIV-1 infected macrophages, which subsequently leads to the stable IL-6 expression and increased HIV-1 replication [103]. Interleukin-6 (IL-6) also reduces the binding of TNF-α to macrophages, which leads to the antagonization of the antimycobacterial effect of TNF-α and, as a result, to a severe course of the TB process [46]. It is believed that IL-6 is a powerful activator of C/EBP (CCAAT-enhancer-binding protein) and that the effect of IL-6 on monocytes also leads to an increase in HIV-1 replication [93,104]. IL-6 hypersecretion promotes viral persistence, disrupting the polarization and functionality of Th1 cells, as well as the lytic ability of CD8+ cells [89]. The reduced concentration of anti-inflammatory cytokines IL-4 and IL-10, which play a protective role in TB and HIV infection, against the background of the elevated levels of cytokine IL-6, which stimulates HIV replication, indicates the progression of both diseases in patients with HIV/TB. The course of tuberculosis associated with HIV infection is characterized by a more severe clinical form of TB, with an increase in the number of infiltrative, fibrous–cavernous, and disseminated forms. It was shown that the increased expression of the IL-6 gene correlates with the delayed dynamics of the resorption of infiltrates in the lung tissue and with the long-term preservation of respiratory complaints in patients during treatment [38]. Quite often, the symptoms of TB associated with HIV infection are non-specific. For this reason, the majority of clinicians note the difficulty of diagnosing tuberculosis in HIV-infected patients, especially at the stage of secondary diseases. As a result, the atypical course of TB in the late stages of HIV infection contributes to the growth of untimely diagnosis of tuberculosis in patients with HIV infection. During X-ray examination, a visible decay in the lung tissue is detected only when necrotic masses are rejected through the bronchi. Therefore, the absence of communication with the bronchus does not indicate the absence of destruction. In our study, 40.3% of patients who were aware of their HIV-positive status and ART-naïve before admission to the hospital were newly diagnosed with TB, and 59.7% were newly diagnosed with HIV and TB. Those patients were admitted to the clinic at the late stages of HIV infection, the course of which was aggravated by undiagnosed and untreated TB. In this regard, early diagnosis of tuberculosis in patients with combined HIV/TB infection is quite urgent. According to the data obtained by us, IL-6 concentrations in patients with HIV-monoinfection and TB-monoinfection against the background of treatment return to normal and become comparable with the control group (on day 75 and day 30, respectively). However, in patients with double HIV/TB infection, IL-6 levels do not decrease and remain enhanced despite the ongoing therapy. Therefore, we believe that an increased persistent level of IL-6 expression in the cohort of HIV/TB patients against the background of ART and reduced concentrations of IL-4 and IL-10 can serve as a possible marker of early detection of *M. tuberculosis* infection in HIV-infected individuals. 

IL-1Ra is a cytokine produced mainly by macrophages and monocytes, as well as neutrophils, fibroblasts, endothelial, and dendritic cells in the acute phase of inflammation [105,106,107]. The mechanism of action of IL-1Ra consists of the blockade of the cellular receptor specific for interleukin-1α and interleukin-1β, as a result of which the activity of the inflammatory cytokine—interleukin-1—is regulated at the site of introduction and replication of viral agents, including the undesirable effects for the body resulting from its excessive production [105,107]. It is known that IL-1Ra plays an extremely important role in the immune response during *Mtb* infection, in particular, in restraining and preventing the spread of infection by the formation of granulomas [108,109,110]. In HIV infection, it has been shown that HIV induces the production of IL-1Ra at the early stages of infection [111,112]. In this study, it was revealed that the plasma level of IL-1Ra in patients with HIV/TB co-infection before the initiation of ART was quite high and significantly exceeded similar indicators in patients only with HIV and only with TB, but already at 45–60 days of therapy, it sharply decreased, and after 6 months of therapy, it reached a level comparable to the same indicator with the control group and two other groups of patients. The data obtained by our group are confirmed by the results of Hoel et al., who showed that patients with HIV/TB co-infection had an increased expression of IL-1Ra before the start of ART and anti-tuberculosis therapy, followed by a decrease in the level of controls, which indicated the success of the therapy [106]. However, a different picture was observed in patients with HIV/TB who developed IRIS associated with TB (TB-IRIS) after 2–3 months of ART. In these patients, the level of IL-1Ra expression was 1.2 times higher than in patients with HIV/TB without TB-IRIS and remained almost at the same level during 6 months of therapy. TB-IRIS is characterized by a paradoxical deterioration of tuberculosis manifestations or even the development of new tuberculosis symptoms in HIV-infected patients after the start of ART, despite the increase in CD4+ cells and decrease in viral load [113,114,115,116,117,118,119]. Usually, TB-IRIS develops within 2–3 months after the start of ART [117,120]. This immunopathological reaction evolves in 4–54% of cases of HIV/TB infection [33,119,120,121,122,123,124,125]. The average duration of TB-IRIS symptoms is about 1–3 months [126,127,128,129], and in some cases, this condition can persist for more than 1 year [33,119,123,124,130,131,132]. In most patients, TB-IRIS disappears spontaneously, without leading to significant mortality. However, in patients, as in this study, who are at the late stages of HIV infection (the median CD4+ cell count was 114 cells/mm^3^), the presence of this syndrome can cause severe local and systemic inflammatory reactions, which in turn can lead to early mortality [133,134,135]. Although it is believed that the main risk factors for the development of TB-IRIS are a low initial level of CD4+ cells and a high initial viral load [121,126,127,128], nevertheless, in this study, the percentage of cases of TB-IRIS was relatively small and amounted to 15.2%, which is consistent with the data of other researchers who identified the frequency of TB-IRIS in patients with HIV/TB co-infection to be also at the level of 11–15% [116,119,136]. There were no cases of fatal outcomes among the studied cohort of HIV/TB patients with TB-IRIS, and after 6 months of therapy, on the whole, this group of patients did not show a significant difference in CD4+ cell growth and a decrease in viral load compared to the group of HIV/TB patients without TB-IRIS. However, the duration and severity of the systemic symptoms (fever with lymph node enlightenment, 43.8%) that developed in patients with TB-IRIS indicate that perhaps the duration of anti-tuberculosis therapy should be prolonged, and the application of ART delayed up to 12 weeks, as recommended by the New York State Department of Health (NYSDOH) AIDS Institute Guidelines [137]. According to the WHO clinical recommendations, ART should be started within 2–8 weeks after the initiation of TB therapy, depending on the number of CD4+ cells [138]. Although the early initiation of ART improves the survival of patients with a low number of CD4+ cells, there is insufficient evidence to confirm or refute the advantage of the early initiation of ART for the survival of HIV/TB patients with a CD4+ cell count >50 cells/mm^3^, but at the same time, the early initiation of ART doubles the frequency of TB-IRIS in this cohort of patients, regardless of the number of CD4+ cells [137,139].

Undoubtedly, this study has certain limitations due to the small number of HIV/TB patients with TB-IRIS. Nevertheless, the high level of IL-1Ra expression (in the range of 1550–2500 pg/mL) even before the start of anti-tuberculosis therapy and ART was detected in HIV/TB patients with newly diagnosed TB who subsequently developed TB-IRIS vs. similar indicator in HIV/TB cohort without IRIS can be considered as a potential predictive biomarker of the development of TB-IRIS and the success of drug therapy at the very beginning of treatment.

In addition, a 4-year follow-up of a cohort of patients with TB monoinfection conducted by Sivro et al. showed that a high level of IL-1Ra and IL-6 expression definitely correlated with TB relapse [140]. Unfortunately, within the framework of this study, we did not have the opportunity to follow patients with HIV/TB for more than 6 months, but we can assume with a high degree of probability that the results obtained by Sivro et al. will also be true for a cohort of patients with HIV/TB co-infection. Moreover, as in the case of elevated IL-6 expression, high levels of IL-1Ra in patients with HIV monoinfection are associated with an increased risk of cardiovascular diseases; in particular, a 1.5-fold increased risk of myocardial infarction [106,141]. Taking this into account, to improve the effectiveness of therapy, physicians should pay attention to the increased level of expression of these cytokines.

## 5. Conclusions

In HIV infection, as the disease progresses, a shift occurs from the secretion of Th1-class cytokines to the secretion of Th2-class cytokines [2,3,63,142]. If at the beginning of the disease, there is an increased expression of Th1 class cytokines, such as IL-2 and IFN-γ, then at later stages, their production decreases, while the production of Th2 class cytokines, IL-4, IL-10 and TNF-α increases [2]. Our study revealed that during a double HIV/TB infection, both Th1 and Th2 immune responses are suppressed. Reduced levels of pro- and anti-inflammatory cytokines in patients with double infection indicate a prolonged dysregulation of the immune response and increased severity of the disease in pulmonary/extrapulmonary tuberculosis in patients with HIV/TB. The monitoring of patients with HIV/TB co-infection in the process of receiving therapy showed that a double HIV/TB infection is characterized by delayed and incomplete recovery of immune activity.

The level of IL-6 secretion in HIV/TB co-infection with newly diagnosed HIV, TB, and previously untreated HIV, both before the initiation of ART and during 6 months of therapy, remained constantly high. Considering that patients with infiltrative pulmonary TB against the background of HIV infection may have elements of destructive decay that are not detected radiologically but are reflected in the immune response, an increased level of IL-6 expression against the background of reduced concentrations of IL-4 and IL-10 can serve as a marker for the early detection of *M. tuberculosis* infection in HIV-infected individuals. The high level of IL-1Ra expression even before the start of anti-tuberculosis therapy and ART detected in HIV/TB patients who subsequently developed TB-IRIS can be considered as a potential predictive biomarker of the development of TB-IRIS and the success of drug therapy at the very beginning of the treatment. Furthermore, the duration and severity of the systemic symptoms (fever with lymph node enlightenment, 43.8%) that developed in patients with TB-IRIS indicate that perhaps the time of anti-tuberculosis therapy should be prolonged and the application of ART delayed by up to 12 weeks. Taking into account the important role that cytokines play in the pathogenesis of HIV infection and TB by regulating innate and adaptive immune responses, the subject of future research is the long-term follow-up (>2 years) of patients with HIV/TB co-infection and the study of the correlation of changes in the immune status with concomitant pathologies.

## Figures and Tables

**Table 1:** Baseline demographic and clinical characteristics of the study population.

Characteristics	HIV/TB (*n* = 89)	HIV (*n* = 80)	TB (*n* = 78)	Healthy Controls (*n* = 82)
**Gender, (*n*/%):**				
Male	58 (65.2)	49 (61.3)	53 (67.9)	56 (68.2)
Female	31 (34.8)	31 (38.7)	25 (32.1)	26 (31.7)
**Age (years), IQR:**				
Male	37.9 (24–72)	37.5 (23–68)	36.9 (28–68)	34.9 (22–48)
Female	38.6 (22–72)	42.3 (22–70)	44.5 (32–71)	37.2 (24–45)
**TB forms, (*n*/%):**				
Disseminated	41 (46.1)		17 (21.8)	
Infiltrative	36 (40.4)		53 (67.9)	
TB of intrathoracic lymph nodes	9 (10.1)		7 (9.0)	
Others	3 (3.4)	_________	1 (1.3)	_________
**CD4+ count (** **cells/mm^3^), %, IQR:**				
<200	71.9%/57 (6–157)	110 (44–189)		
<350	19.1%/215 (203–310)	272 (222–342)		
>350	9%/352 (352–380)	553 (383–711)	487 (379–520)	978 (800–1200)
**Viral load (log_10_ copies/mL), IQR**	6.8 (1.9–7.1)	6.2 (1.5–6.5)	__________	_________

IQR—interquartile range.

**Table 2:** Symptoms in the HIV/TB patients during development of IRIS (*n* = 16).

Symptoms	Frequency
Fever only	2 (12.5%)
Fever with lymph node enlargement	7 (43.8%)
Fever with alveolar pneumonitis	2 (12.5%)
Fever with abdominal lymphadenopathy	2 (12.5%)
Abdominal lymphadenopathy only	1 (6.25%)
Lymph node enlargement only	2 (12.5%)

**Table 3:** Characteristics of patients with HIV/TB co-infection with and without IRIS.

Characteristics	IRIS Neg (*n* = 89)	IRIS Pos (*n* = 16)	*p*-Value
**TB forms (%/*n*):**			
Pulmonary	79.7 (71)	68.8 (11)	0.0001
Extrapulmonary	10.1 (9)	12.5 (2)	
**CD4+ count (cells/mm^3^), IQR:**			
Before therapy	114 (7–380)	112 (5–357)	0.9772
After therapy (180 days)	393 (66–703)	381 (40–509)	0.7233
**Viral load (log_10_ copies/mL), IQR:**			
Before therapy	6.8 (1.9–7.01)	5.5 (1.9–6.1)	0.5967
After therapy (180 days)	1.7 (1.2–1.9)	1.7 (1.3–1.9)	0.9023

IQR—interquartile range.

## Data Availability

Data available upon request from the authors.
